# Prediction of heptagonal bipyramidal nonacoordination in highly viable [OB-M©B_7_O_7_-BO]^−^ (M = Fe, Ru, Os) complexes

**DOI:** 10.1038/s42004-021-00620-0

**Published:** 2022-01-10

**Authors:** Bo Jin, Hai-Ru Li, Zhihong Wei, Miao Yan, Caixia Yuan, Yan-Bo Wu, Si-Dian Li

**Affiliations:** 1 Key Laboratory of Materials for Energy Conversion and Storage, Key Laboratory of Chemical Biology and Molecular Engineering of Ministry of Education, Institute of Molecular Science, Shanxi University, Taiyuan, Shanxi PR China; 2 School of Energy and Power Engineering, North University of China, Taiyuan, Shanxi PR China

**Keywords:** Coordination chemistry, Structure prediction

## Abstract

Non-spherical distributions of ligand atoms in coordination complexes are generally unfavorable due to higher repulsion than for spherical distributions. To the best of our knowledge, non-spherical heptagonal bipyramidal nonacoordination is hitherto unreported, because of extremely high repulsion among seven equatorial ligand atoms. Herein, we report the computational prediction of such nonacoordination, which is constructed by the synergetic coordination of an equatorial hepta-dentate centripetal ligand (B_7_O_7_) and two axial mono-dentate ligands (-BO) in the gear-like mono-anionic complexes [OB-M©B_7_O_7_-BO]^–^ (M = Fe, Ru, Os). The high repulsion among seven equatorial ligand B atoms has been compensated by the strong B–O bonding. These complexes are the dynamically stable (up to 1500 K) global energy minima with the HOMO-LUMO gaps of 7.15 to 7.42 eV and first vertical detachment energies of 6.14 to 6.66 eV (being very high for anions), suggesting their high probability for experimental realization in both gas-phase and condensed phases. The high stability stems geometrically from the surrounded outer-shell oxygen atoms and electronically from meeting the 18e rule as well as possessing the σ + π + δ triple aromaticity. Remarkably, the ligand-metal interactions are governed not by the familiar donation and backdonation interactions, but by the electrostatic interactions and electron-sharing bonding.

## Abstract (web summary)

Complexes possessing coordination spheres that can accommodate nine ligand atoms typically display spherical distributions of these atoms. Here, the authors predict that M-centered [OB-B_7_O_7_-BO] adopts unusual heptagonal bipyramidal nonacoordination.

## Introduction

**Fig. 1:**
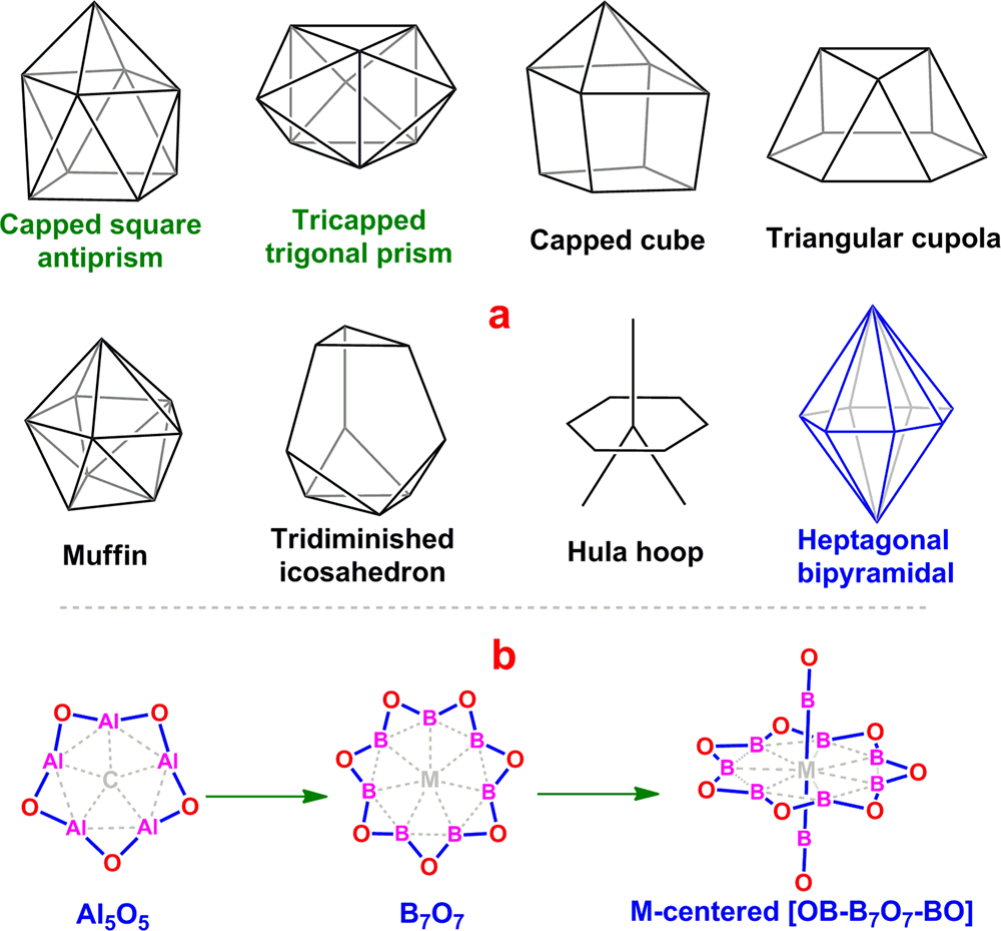
Configurations mentioned in introduction section. **a** The possible configurations of nonacoordination. **b** How the heptagonal bipyramidal configuration was come up with.

The arrangement of the ligands around an atom generally complies with the valence shell electron pair repulsion (VSEPR) theory^1,2^. In precise, the arrangements for two and three valence shell electron pairs (VSEPs) are commonly linear and planar triangular, respectively, while those for higher numbers of VSEPs are all three-dimensional^3–5^. Remarkably, when the number (*n*) of VSEPs increases, the distribution of VSEPs tends to be spherical, which can minimize the repulsion among ligand atoms. This is also true for the metal complexes, especially when *n* is higher than 6. Alvarez et al. had surveyed a series of complexes with 7 to 10 vertexes in coordination polyhedrons and the spherical distribution of the ligands was usually found^6–9^. Specifically, the complexes with nonacoordination, commonly emerged for the lanthanide (Ln), also majorly adopt the spherical distributions of ligand atoms, as typified by the capped square antiprism or tricapped trigonal prism (Fig. 1a). In contrast, due to the higher strains originated from the repulsions among the ligand atoms, it appears to be hard to achieve the nonspherical configurations (see Fig. 1a), such as the capped cube, triangular cupola, muffin, tridiminished icosahedron, hula hoop, and heptagonal bipyramidal^10^.

In this work, we focus on the highly symmetric heptagonal bipyramidal configuration, which is unknown to date in the literature, both computationally and experimentally. To construct the heptagonal bipyramidal configuration, it needs the synergetic coordination of seven ligand atoms in the equatorial plane and two ligand atoms in the directions perpendicular to such plane. The difficulty in achieving the heptagonal bipyramidal configuration mainly comes from the unimaginably high repulsion among seven ligand atoms in the equatorial plane. Can such repulsion be overcome so that the heptagonal bipyramidal configuration is favorable? The answer is positive. In this work, we report the [OB-M©B_7_O_7_-BO]^−^ (M = Fe, Ru, Os) complexes, where the repulsion among seven equatorial boron atoms is compensated by the strong B–O bonding, leading to the higher preference of heptagonal bipyramidal configuration over the spherical configurations.

## Results and discussion

### Structures of [OB-M©B_7_O_7_-BO]^−^ (M = Fe, Ru, Os)

The present work is inspired by our recently designed planar pentacoordinate carbon (ppC) species CAl_5_O_5_^+^^11^, where the Al_5_O_5_ moiety is arranged in a zig-zag configuration (Fig. 1b) and serves as a planar penta-dentate centripetal five-electron donor. Moreover, five Al atoms are strongly bonded together by five bridging O atoms through ten Al–O two-center two-electron (2c–2e) σ bonds and five Al–O–Al three-center two-electron (3c–2e) π bonds, leading to the higher preference of the planar ppC structure (the global energy minimum) over the three-dimensional (3D) structures. Such a bonding manner stimulates us: A proper zig-zag-arranged E_7_O_7_ moiety may be suitable to serve as a hepta-dentate centripetal ligand for designing the complexes having the desired the heptagonal bipyramidal configuration. Since the negative ion photoelectron spectroscopy (PES) is one of the most powerful tools to characterize the exotic structures in the gas-phase, the ternary mono-anionic complexes are preferred to facilitate their future experimental realizations.

**Fig. 2:**
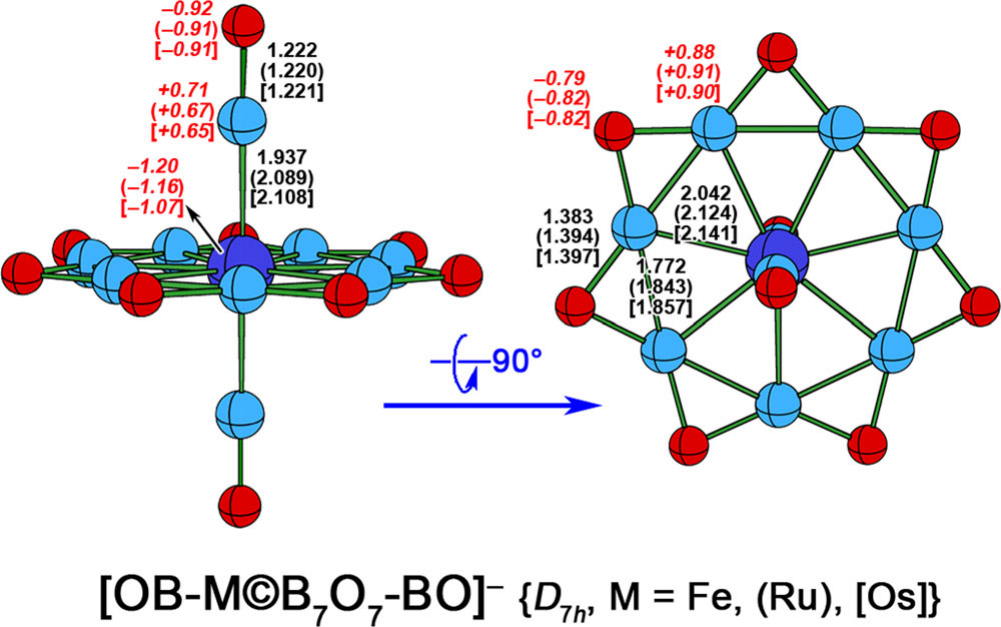
Side (left) and top (right) views of *D*_*7h*_ [OB-M©B_7_O_7_-BO]^−^ (M = Fe (1), Ru (2), Os (3)) at the PBE0/BS1 level. The necessary bond distances are indicated in Å and the natural charges (in red italic font) are indicated in |e|.

Along the lines of these thoughts, we first examined the feasibility of the Al_7_O_7_ ring as the equatorial ligand and two –AlO groups as the axial ligands to construct the heptagonal bipyramidal configuration, but the Al_7_O_7_ ring was too big in size for any transition metal to stay inside. Then, we considered the smaller B_7_O_7_ ring (Fig. 1b) in combination with two boronyl (–BO) ligands^12,13^. By complying with the 18e rule, such a combination matches a group 8 metal (Fe, Ru, Os) to give a mono-anionic complex with heptagonal bipyramidal configuration for central metal atom (Fig. 1b). Delightfully, we had turned out that the [OB-M©B_7_O_7_-BO]^−^ (M = Fe (**1**), Ru (**2**), Os (**3**), see Fig. 2) complexes were all the energy minima (equilibrium structures) adopting the high symmetry of *D*_7*h*_ at the PBE0/BS1 level, where BS1 denotes a mixed basis set with cc-pVTZ for B, O, and Fe, and cc-pVTZ-PP for Ru and Os.

As shown in Fig. 2, the equatorial/axial B-M distances are 2.042/1.937, 2.124/2.089, and 2.141/2.108 Å for M = Fe, Ru, and Os, respectively, which are very close to the sum of single-bond covalent radii of B and corresponding M atoms (2.01, 2.10, and 2.14 Å). Therefore, nine B atoms in **1**–**3** can be counted as the coordinations to the M center, i.e., the M atom in **1**–**3** adopts the desired heptagonal bipyramidal configuration. Interestingly, though the equatorial B–O distances are relatively rigid, as reflected by the gent elongation from 1.383 to 1.397 Å when M goes from Fe to Os, the equatorial B–B distances show the flexibility, as mirrored by the obvious elongation from 1.772 to 1.857 Å under the same variation of M atoms. The flexible B–B distances may suggest the absence of direct localized 2c–2e B–B bonds. The axial B–O distances range from 1.220 to 1.222 Å, indicating the formation of B–O multiple bonds.

### Stability consideration

Topologically, the chemically active B atoms are all protected by the oxygen atoms in **1**–**3**, possibly leading to high stability. In this work, the thermodynamic stability was studied by extensive exploration of potential energy surfaces of [OB-M©B_7_O_7_-BO]^−^ (M = Fe, Ru, Os) components using basin hopping algorithm. The relative energies (ΔE) of isomers were finally determined at the CCSD(T)/BS1 level considering the zero-point energy (ZPE) obtained at the PBE0/BS1 level (abbreviated as CCSD(T) + ZPE_PBE0_, see the details of computation in Computational Methods section). **1**–**3** are all confirmed to be the global energy minima on corresponding MB_9_O_9_^−^ potential energy surfaces, which are 13.2, 9.5, and 8.2 kcal mol^−1^ lower in energy than the second lowest lying isomers (Supplementary Fig. S1), respectively.

**Fig. 3:**
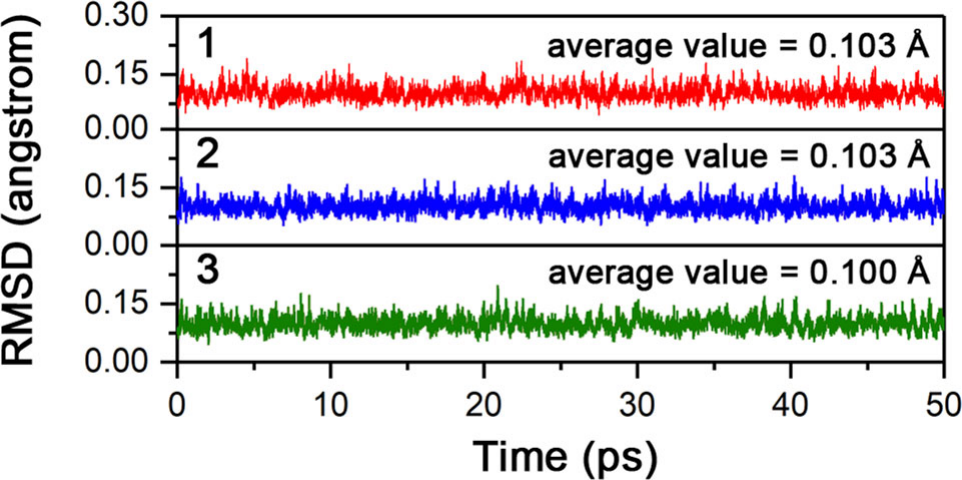
Results of BOMD simulations for 1-3. RMSD (in Å) versus simulation time (in ps) for the BOMD simulations of [OB-M©B_7_O_7_-BO]^−^ (M = Fe, Ru, Os, 1–3) at the PBE/DZVP level and 1500 K.

The dynamic stability of **1**–**3** was studied using 50 ps Born–Oppenheimer molecular dynamic (BOMD) simulations at the PBE/DZVP level and the concerned temperatures (4, 298, 500, 1000, and 1500 K). As shown in Fig. 3, **1**–**3** well maintain their basic structures at 1500 K, as reflected by the plots of root-mean-square deviation (RMSD, relative to PBE/DZVP-optimized structures) without significant upward jumps. The fluctuation of the RMSD values is small, as mirrored by the average values of 0.103, 0.103, and 0.100 Å with the variation ranges of 0.044–0.192, 0.052–0.180, and 0.046–0.196 Å for **1**, **2**, and **3**, respectively. The BOMD simulations indicate that **1**–**3** possess the high dynamic stability. The RMSD plots for the simulations at lower temperatures are given in Supplementary Figs. S2–S4.

The high chemical stability of **1**–**3** can be well reflected firstly by their calculated large HOMO-LUMO gaps of 7.15, 7.30, and 7.42 eV at the PBE0/BS1 level, respectively. It can be further supported by the high first vertical detachment energies (VDEs) of 6.66, 6.14, and 6.25 eV for **1**, **2**, and **3**, respectively, at the CCSD(T)/BS1 level. Such high VDEs offer the fingerprints for spectroscopic studies (see the simulated PES in Supplementary Fig. S5). Moreover, the unusually large HOMO-LUMO gaps and high VDEs suggest that **1**–**3** may be synthesizable not only in the gas phase, but also in the condensed phase (as the negative portion of an ionic salt).

### Electronic structure analysis

**Fig. 4:**
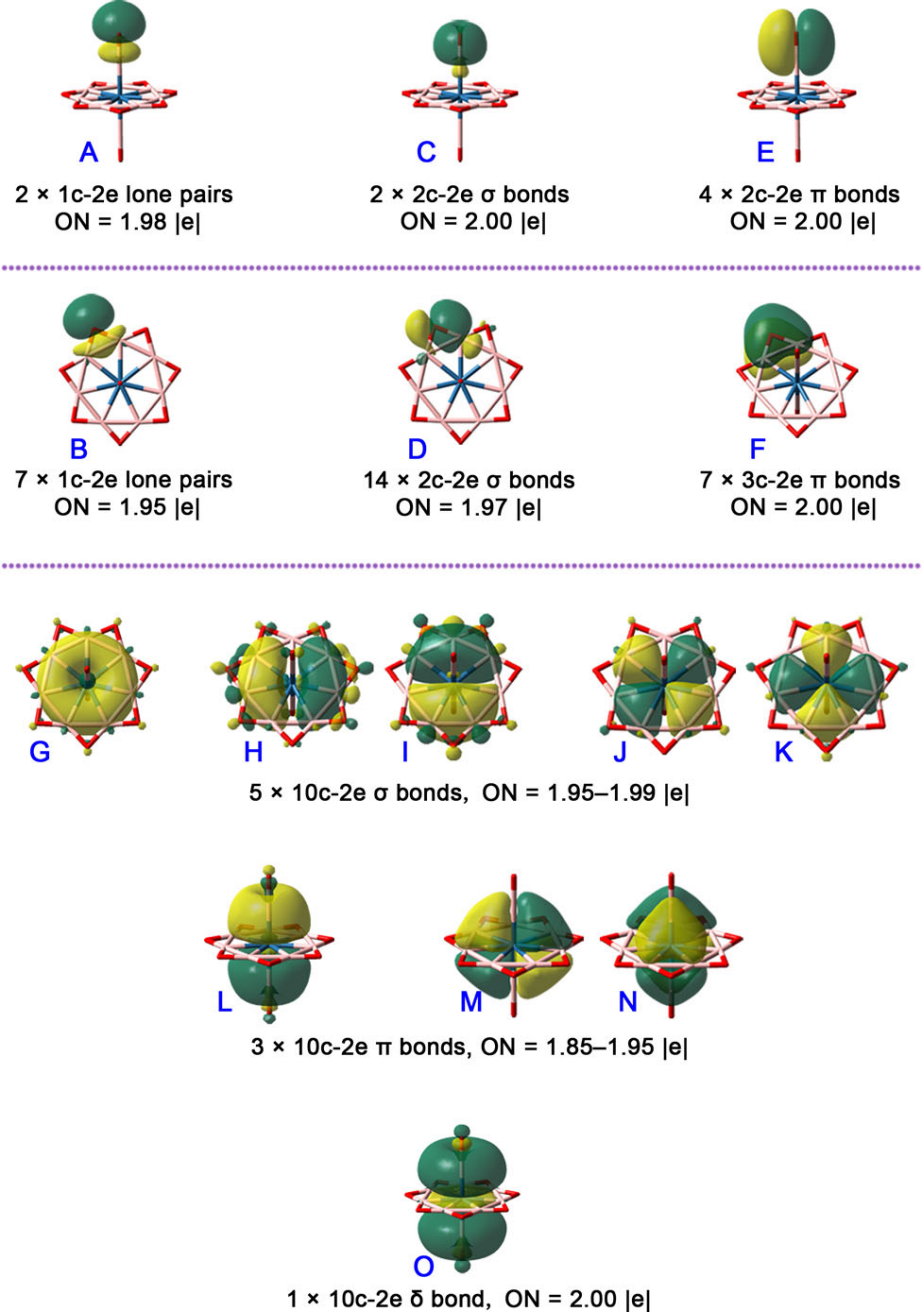
AdNDP bonding patterns of [OB-Os©B_7_O_7_-BO]^−^ (3). Only one orbital is shown when multiple orbitals in a pattern are identical due to molecular symmetry.

To interpret the high electronic stabilities of **1**–**3**, detailed adaptive natural density partitioning (AdNDP) bonding analyses are performed. AdNDP approach provides a clear bonding scheme for a system in the form of *n*-center two-electron (*n*c–2e) bonds, where *n* can range from 1 to the total number of atoms in a molecule. Shown in Fig. 4 are the shapes of partitioned orbitals of **3**. In forty-five valence electron pairs in **3**, eight pairs are within two axial BO groups, twenty-eight pairs concern the B–O bonding in the equatorial B_7_O_7_ group, and nine pairs concern the bonding within central B_9_Os moiety. Specifically, each of the two axial BO ligands possesses one 1c–2e lone pair of O (orbital shape **A**) with an occupation number (ON) of 1.98|e|, one B–O 2c–2e σ bonds (**B**, ON = 2.00|e|), and two B–O 2c–2e π bonds (**C**, ON = 2.00|e|). The equatorial B_7_O_7_ ligand possesses seven 1c–2e lone pairs of O atoms (**D**, ON = 1.95|e|), fourteen B–O 2c–2e σ bonds (**E**, ON = 1.97 |e|), and seven B–O–B 3c–2e π bonds (**F**, ON = 2.00 |e|). Though seven boron atoms are densely arranged in a plane, the AdNDP orbitals clearly show that the bridging O atoms strongly bound them together *via* the B–O σ bonds and B–O–B π bonds, which can effectively compensate the repulsion among seven boron atoms. The remaining nine orbitals are delocalized 10c–2e bonds over the B_9_Os moiety, fully filling the valence shells of Os atom and meeting the 18e rule. Remarkably, the aromaticity, especially the exotic type of aromaticity, often plays the important role in stabilizing the exotic structures^14–16^. For **3**, if the B_7_Os plane is taken as the reference to justify the orbital symmetries, there are five (**G**–**K**), three (**L**–**N**), and one (**O**) delocalized bonds in σ, π, and δ symmetries with ONs of 1.95–1.99, 1.85–1.95, and 2.00|e|, respectively. The numbers of delocalized electrons in σ, π, and δ systems are therefore 10, 6, and 2, matching the Hückel’s 4*n* + 2 aromatic rule with *n* = 2, 1, and 0, respectively. Such a bonding pattern renders a unique σ + π + δ triple aromaticity to **3**. Since both **1** and **2** are similar to **3** in AdNDP bonding patterns, they are given in Supplementary Figs. S6, S7.

### Aromaticity

**Fig. 5:**
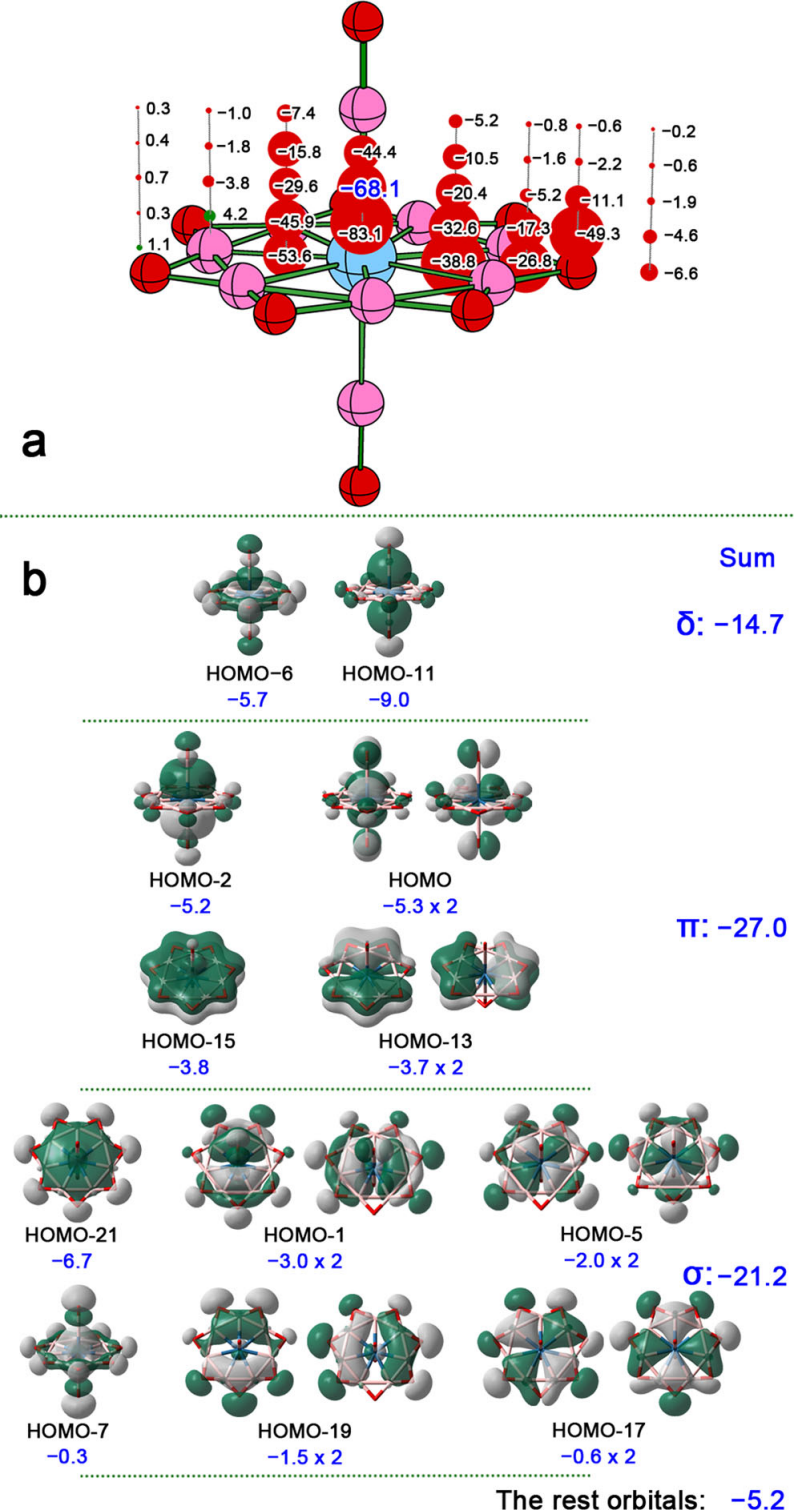
NICS results of 3. **a** The calculated NICS values for the ghost atoms located in a symmetry plane (the positive and negative NICS values are shown in green and red bare balls, respectively). **b** The dissected contributions from individual canonical molecular orbitals to the total NICS value for the point located 1 Å above the center Os atom.

The aromaticity in **1**–**3** can be proved by nucleus-independent chemical shift (NICS) calculations. The results for **3** are shown in Fig. 5, while those for **1** and **2** are given in the Supplementary Figs. S8, S9. For NICS calculations, the examined points distribute in a symmetry plane. As the Fig. 5a shows, except for the points locate 0.5 Å above the boron atoms, all other points within or above the B_7_O_7_ moiety possess the negative NICS values, suggesting the aromatic electron current. In particular, the points within the B_9_ heptagonal bipyramidal polyhedron possess the obvious negative NICS values (ranging from −32.6 to −83.1 ppm), pronouncing the great contributions from the nine delocalized ligand–metal bonding orbitals. Remarkably, the total NICS value for each examined point can be dissected into the contributions from the individual canonical molecular orbitals (CMOs). Taking the point located 1 Å above the Os atom with the NICS value of −68.1 ppm as an example, 92.4% contributions (−62.9 ppm) for total NICS come from the CMOs shown in Fig. 5b. These CMOs can be seen as the linearly combined orbitals of the AdNDP-partitioned orbitals (**G**–**O**) shown in Fig. 4 and other orbitals concerning O lone pairs or B–O–B 3c–2e π bonds. Specifically, HOMO-6 and HOMO-11 have a similar δ symmetry to AdNDP orbital **O**, giving the contribution of −14.7 ppm. HOMO (degenerate), HOMO-2, HOMO-13 (degenerate), and HOMO-15 have the alike π symmetry to AdNDP orbitals **L**–**N**, leading to the contribution of −27.0 ppm. Degenerate HOMO-1, degenerate HOMO-5, HOMO-7, degenerate HOMO-17, degenerate HOMO-19, and HOMO-21 have the parallel σ symmetry to AdNDP orbitals **G**–**K**, causing the contribution of −21.2 ppm. In summary, the delocalized δ, π, and σ orbitals account for 21.6%, 39.6%, and 31.1% of total NICS value, so **3** is triply aromatic. Please note that the contributions from these eighteen CMOs are actually the contributions of nine AdNDP orbitals **G**–**O**, so the number of delocalized orbitals in δ, π, and σ symmetries are still 1, 3, and 5, respectively.

### Ligand–metal interactions

We further performed the energy decomposition analysis–natural orbitals for chemical valence (EDA-NOCV) to deepen the understanding of metal–ligand bonding. Given in Table 1 are the results concerning the scheme with the smallest orbital interaction energies^17^. As the Table 1 shows, the Pauli repulsion energies (Δ*E*_Pauli_) are high and increase dramatically as M goes from Fe to Os (from 518.9 to 1071.3 kcal mol^–1^), which is highly related to the increasing number of electrons in M atoms because the Pauli repulsion originates from the nature of electron (as a Fermion). Nevertheless, the total attractive energies (Δ*E*_Attr_) also increase dramatically (from −1054.6 to −1758.3 kcal mol^–1^, respectively), leading to the increasing interaction energies (Δ*E*_int_) of −535.7, −591.3, and −687.0 kcal mol^–1^ for **1**, **2**, and **3**, respectively and indicate the very strong intrinsic attraction between M center and [OB-B_7_O_7_-BO]^−^ moiety. Notably, the Δ*E*_Attr_ has the higher electrostatic (Δ*E*_elstat_) portions (54.3–56.8%) than covalent (Δ*E*_orb_) portions (43.2–45.7%).

**Table 1:** Results of EDA-NOCV calculations for singlet state [OB-M©B_7_O_7_-BO]^−^ complexes at the PBE0/TZ2P level using neutral M atoms in the quintet reference state with *n*s^0^*n*p^1^(*n* − 1)d^7^ electron configuration and [OB-B_7_O_7_-BO]^−^ (L, quintet) as interacting fragments.

Energy terms	Number of orbitals	Assignments	Interaction energies for M (quintet, *n*s^0^*n*p^1^(*n* − 1)d^7^) + [OB-B_7_O_7_-BO]^−^ (L, quintet)		
			M = Fe	M = Ru	M = Os
∆*E*_int_			−535.7	−591.3	−687.0
∆*E*_Pauli_			518.9	746.5	1071.3
∆*E*_Attr_			−1054.6	−1337.8	−1758.3
∆*E*_elstat_^[a]^			−592.6 (56.2%)	−760.0 (56.8%)	−953.9 (54.3%)
∆*E*_orb_^[a]^			−462.0 (43.8%)	−577.8 (43.2%)	−804.4 (45.7%)
∆*E*_orb(1)_^[b]^ (A_1_′)	1	L-M(d_z2_) electron-sharing bonds	−97.6 (21.1%)	−128.2 (22.2%)	−305.5 (38.0%)
∆*E*_orb(2)_^[b]^ (E_2_′)	2	L-M(d_xy_/d_x2–y2_) electron-sharing bonds	−195.6 (42.3%)	−274.4 (47.5%)	−280.4 (34.9%)
∆*E*_orb(3)_^[b]^ (E_1_″)	1	L-M(p_z_) electron-sharing bonds	−46.7 (10.1%)	−50.3 (8.7%)	−53.8 (6.7%)
∆*E*_orb(4)_^[b]^ (A_2_″)	2	L ← M(d_xz_/d_yz_) backdonation	−94.0 (20.3%)	−90.2 (15.6%)	−123.6 (15.4%)
∆*E*_orb(5)_^[b]^ (E_1_′)	2	L → M(p_x_/p_y_) donation	−15.4 (3.3%)	−15.8 (2.7%)	−16.8 (2.1%)
∆*E*_orb(6)_^[b]^ (A_1_′)	1	L → M(s) donation	−4.8 (1.0%)	−7.6 (1.3%)	−12.0 (1.5%)
∆*E*_rest_^[b]^			−7.9 (1.7%)	−11.3 (2.0%)	−12.3 (1.5%)

Energy values are given in kcal mol^−1^.

^[a]^The percentage values in the parenthesis are the contribution to the total attractive interactions (∆*E*_elstat_ + ∆*E*_orb_).

^[b]^The percentage values in the parenthesis are the contribution to the total orbital interactions (∆*E*_orb_).

The total Δ*E*_orb_ can be further dissected into contributions from individual orbitals. As shown in Table 1, the orb(1)–orb(3) concerning the electron-sharing bonding characters play the dominant role in Δ*E*_orb_ (73.5–79.6%). These orbitals can be associated with the d_z2_, d_xy_/d_x2–y2_, and p_z_ AOs of the metal. The much less roles were played by the degenerate orbital [orb(4)] featuring the metal-to-ligand (M → L) backdonation characters and concerning d_xz_/d_yz_ AOs of the metal (15.4–20.3%). As a comparison, the orbitals [orb(5) and orb(6)] feature the ligand-to-metal (L → M) donation characters and concern the p_x_/p_y_ and s AOs of the metal, which contribute almost negligible to the covalent bonding (3.6–4.3%).

**Fig. 6:**
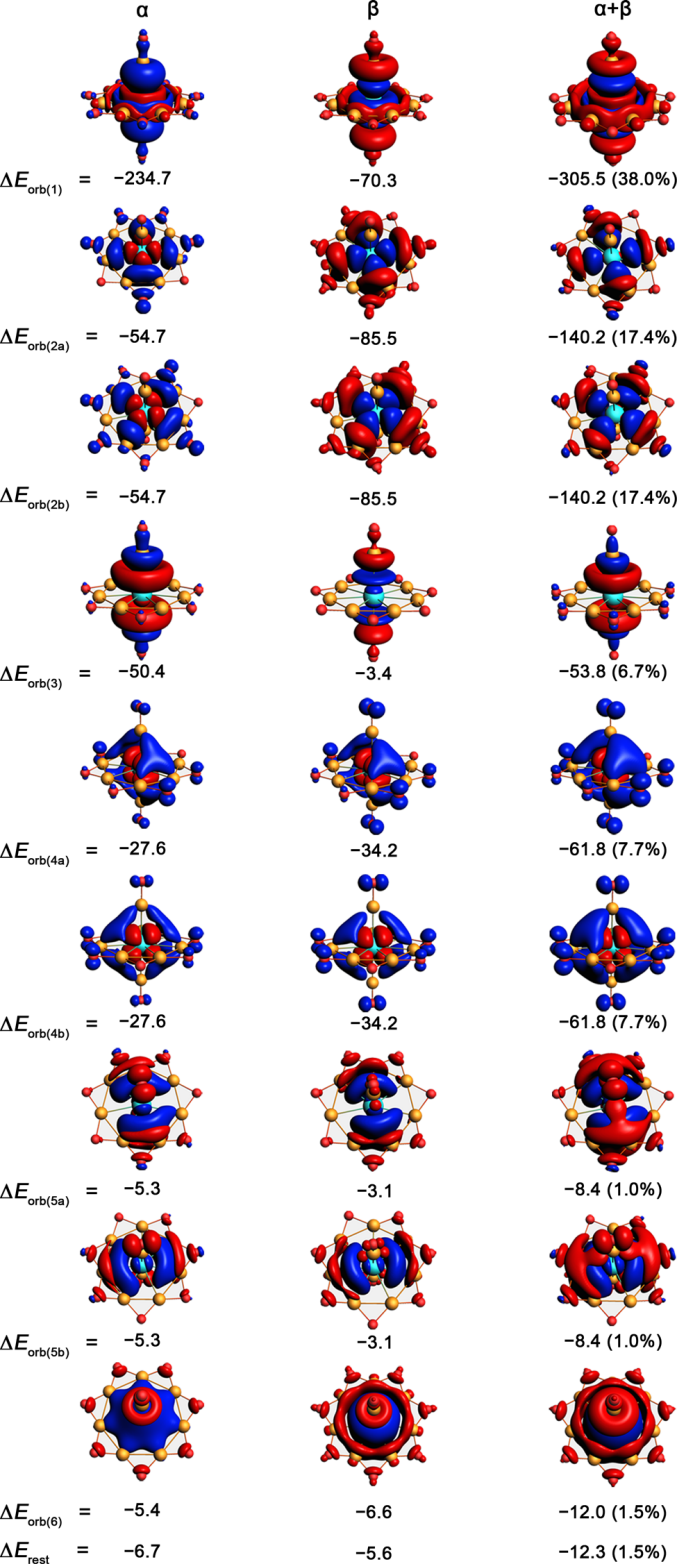
The shapes of deformation densities (Δ*ρ*) for EDA-NOCV analysis of 3. Both the related molecular orbitals and corresponding α and β spin orbitals are shown. The isovalues of the surfaces are 0.001 for Δ*ρ*_orb(1)–(4)_, 0.0003 for Δ*ρ*_orb(5)_ and 0.0002 for Δ*ρ*_orb(6)_. The direction of charge flow is from red to blue.

Figure 6 shows the deformation densities (Δ*ρ*) of **3** connecting to the concerned orbitals, while the Δ*ρ* results of **1** and **2** are similar to those of **3**, thus they are given in Supplementary Figs. S10, S11. As the Fig. 6 shows, the α and β spin orbitals of orb(1)–(3) have the opposite charge flow, demonstrating their electron-sharing nature, while those of orb(4)–(6) are uniform, in line with their dative nature (L → Os donation or Os→L backdonation). Since there are three orbitals featuring L → M donation characters, which is higher than that (two orbitals) featuring M → L backdonation characters, the whole charge flow in **3** shows obviously LMCT (ligand to metal charge transfer) characters. This can be verified by the natural population analysis (NPA), which locates the negative charges of −1.20, −1.16, and −1.07 | e| on central M atoms for M = Fe, Ru, and Os, respectively (see Fig. 2). Following the suggestion of our reviewer, we also performed the orbital correlation analysis to further confirm the natures of these orbitals and the results are given in Supplementary Figs. S12–S14. As the figures show, the orbitals concerning the d_xy_/d_x2–y2_, d_z2_, and p_z_ are electron-sharing bonds, while other orbitals are dative bonds, related to either L → M donation or M → L backdonation.

## Conclusions

In summary, we have computationally demonstrated that the heptagonal bipyramidal configuration can be achieved by synergetic coordination of an equatorial hepta-dentate centripetal ligand (B_7_O_7_) and two axial mono-dentate ligands (–BO) to a group 8 transition metal in gear-like complexes [OB-M©B_7_O_7_-BO]^−^ (M = Fe, Ru, Os). We have also demonstrated and rationalized their excellent thermodynamic, dynamic, and electronic stabilities, which should enable their experimental realization and characterization. The ligands, i.e., the zig-zag B_*n*_O_*n*_ ring and boronyl (–BO), may be useful in the realization of other non-classical coordination stereochemistry.

### Computational methods

Geometry optimization and harmonic vibrational frequency analysis of **1**–**3** were performed at the PBE0/BS1 level, where BS1 denotes a mixed basis set with cc-pVTZ for B, O, and Fe, and cc-pVTZ-PP for Ru^18^ and Os^19^. AdNDP^20^ analyses were performed at the PBE0/BS2 levels, where BS2 denotes a mixed basis set with 6-31G(d) for B, O, and Fe, and SDD for Ru and Os, respectively. The thermodynamic stability was studied by the exploration of potential energy surfaces of **1**–**3** using basin hopping algorithm^21^. The seeds for the exploration came from manually constructed structures corresponding to the configurations shown in Fig. 1a and some structures obtained using the stochastic search algorithm^22–25^. The located isomers were initially optimized at the PBE^26^/DZVP^27^ level and then the 20 low-energy isomers re-optimized at the PBE0/BS1 level. The single point energies of the five lowest isomers were calculated at the CCSD(T)^28^/BS1 level and corrected with the PBE0/BS1 zero-point energies (Supplementary Fig. S1), which is abbreviated as CCSD(T) + ZPE_PBE0_ and reported in the text. The dynamic stability of **1**–**3** was studied using BOMD simulation at the PBE/DZVP level for 50 ps using the CP2K package^29^. Nucleus-independent chemical shifts (NICS)^30–33^ were calculated to assess the aromaticity of **1**–**3**. The basin hopping algorithm was realized using the Tsinghua Global Minimum (TGmin) program^34^, the CCSD(T) calculations were carried out using the MolPro 2012.1 package^35^, the dissected contributions of individual CMOs to NICS values are analysis using NBO 6.0 package^36^, the EDA-NOCV^37,38^ were performed at the PBE0/TZ2P^39^ level using the ADF 2019 program package^40^, and all other calculations were performed using the Gaussian 16 package^41^.

## Supplementary information

Supplementary Information

Supplementary Data 1

Description of Additional Supplementary Files

## Data Availability

The authors declare that all other data supporting the findings of this study are available within the paper, its Supplementary Information, and Supplementary Data File. Furthermore, Supplementary Data 1 includes all Cartesian coordinates of structures in Fig. 2 and Supplementary Fig. S1 as well as that of the initial and final configurations for dynamics trajectories.
