# Supplementary material for: Prediction of heptagonal bipyramidal nonacoordination in highly viable [OB-M©B7O7-BO]− (M = Fe, Ru, Os) complexes
Source: Commun Chem. 2022 Jan 10;5:1. doi: 10.1038/s42004-021-00620-0 (PMC9814638; doi:10.1038/s42004-021-00620-0)
Supplement: Supplementary file 1 — Supplementary Information [file 42004_2021_620_MOESM1_ESM.pdf]

Supplementary Information

Prediction of heptagonal bipyramidal nonacoordination in highly viable $[OB\text{-}M©B_7O_7\text{-}BO]^-$ (M = Fe, Ru, Os) complexes

Bo Jin,[1] Hai-Ru Li,[1,2] Zhihong Wei,[1] Miao Yan,[1] Caixia Yuan,[1] Yan-Bo Wu,[1,*] Si-Dian Li[1,*]

1. Key Laboratory of Materials for Energy Conversion and Storage, Key Laboratory of Chemical Biology and Molecular Engineering of Ministry of Education, Institute of Molecular Science, Shanxi University, Taiyuan, Shanxi 030006, People's Republic of China.
2. School of Energy and Power Engineering, North University of China, Taiyuan, Shanxi 030051, People's Republic of China.

*To whom correspondence should be addressed.

E-mail: wyb@sxu.edu.cn (Y.B.W.) and lisidian@sxu.edu.cn (S.D.L.)

Contents

Supplementary Figure S1. Structures and relative energies (ΔE, in kcal mol^{-1} at the CCSD(T) + ZPE_{PBE0} level) of $[OB\text{-}M©B_7O_7\text{-}BO]^-$ (M = Fe, Ru, Os) and their low-energy isomers.

Supplementary Figure S2–S4. RMSD (in Å) versus simulation time (in ps) for the BOMD simulation of $[OB\text{-}M©B_7O_7\text{-}BO]^-$ (M = Fe, Ru, Os) at the PBE/DZVP level. The examined temperatures include 4, 298, 500, and 1000 K.

Supplementary Figure S5. The simulated PES spectra of $[OB\text{-}M©B_7O_7\text{-}BO]^-$ (M = Fe, Ru, Os).

Supplementary Figure S6–S7. AdNDP bonding patterns of $[OB\text{-}M©B_7O_7\text{-}BO]^-$ (M = Fe, Ru).

Supplementary Figure S8–S9. NICS results of 1–2.

Supplementary Figure S10–S11. The shapes of deformation densities (Δ*ρ*) for EDA-NOCV analysis of 1–2

Supplementary Figure S12–S14. MO correlation diagram of Fe, Ru, and Os with $[OB\text{-}B_7O_7\text{-}BO]^-$ ligand.

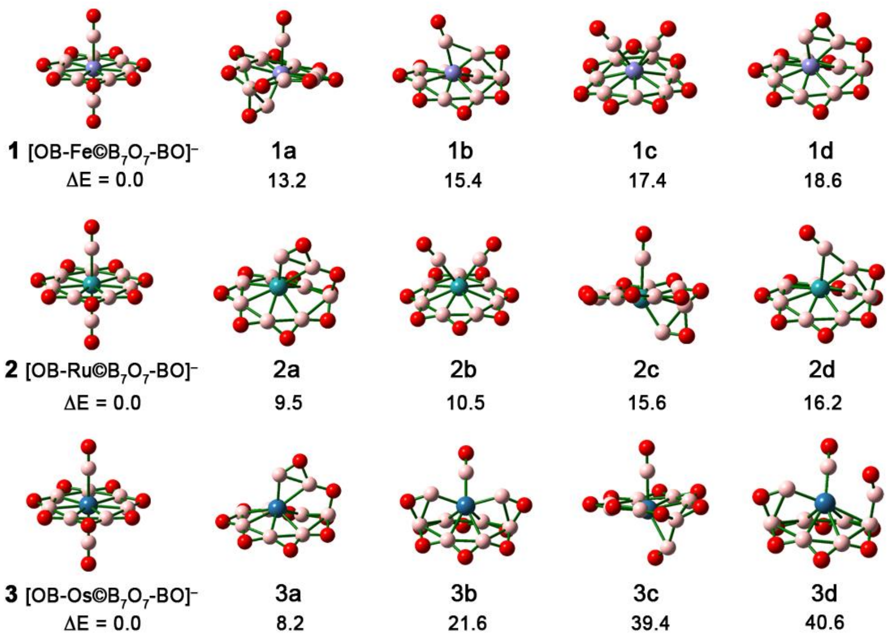

Supplementary Figure S1. Structures and relative energies (ΔE, in kcal mol^{-1} at the CCSD(T) + ZPE$_{PBE0}$ level) of [OB-M©B_7O_7-BO]$^-$ (M = Fe, Ru, Os, 1–3) and their low-energy isomers.

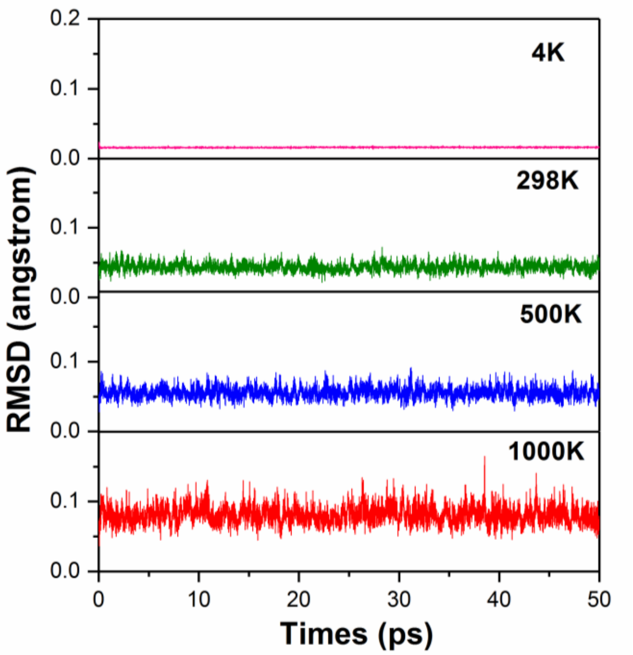

Supplementary Figure S2. RMSD (in Å) versus simulation time (in ps) for the BOMD simulation of [OB-Fe©B_7O_7-BO]$^-$ at the PBE/DZVP level. The examined temperatures include 4, 298, 500, and 1000 K.

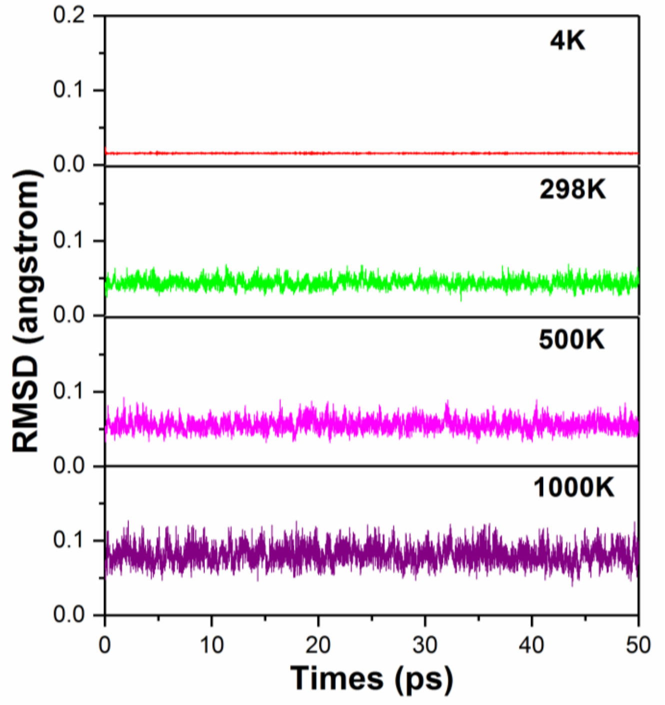

Supplementary Figure S3. RMSD (in Å) versus simulation time (in ps) for the BOMD simulation of $[OB\text{-}Ru©B_7O_7\text{-}BO]^-$ at the PBE/DZVP level. The examined temperatures include 4, 298, 500, and 1000 K.

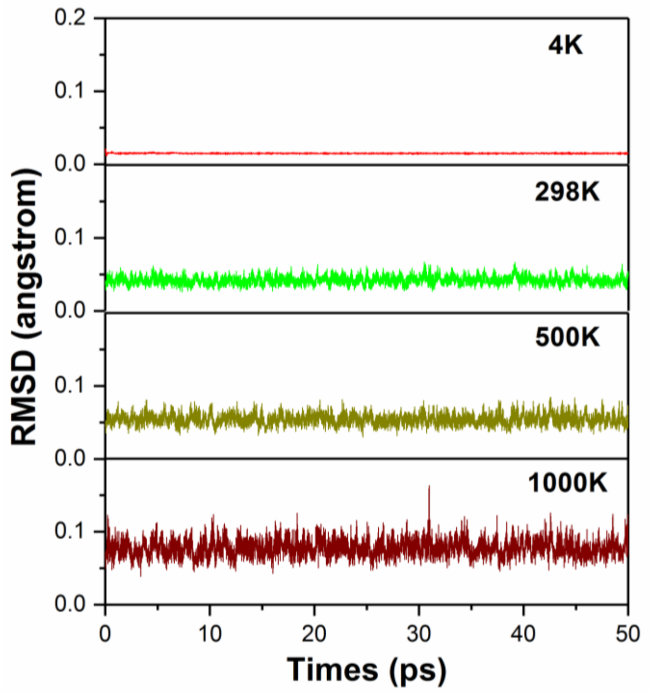

Supplementary Figure S4. RMSD (in Å) versus simulation time (in ps) for the BOMD simulation of $[OB\text{-}Os©B_7O_7\text{-}BO]^-$ at the PBE/DZVP level. The examined temperatures include 4, 298, 500, and 1000 K.

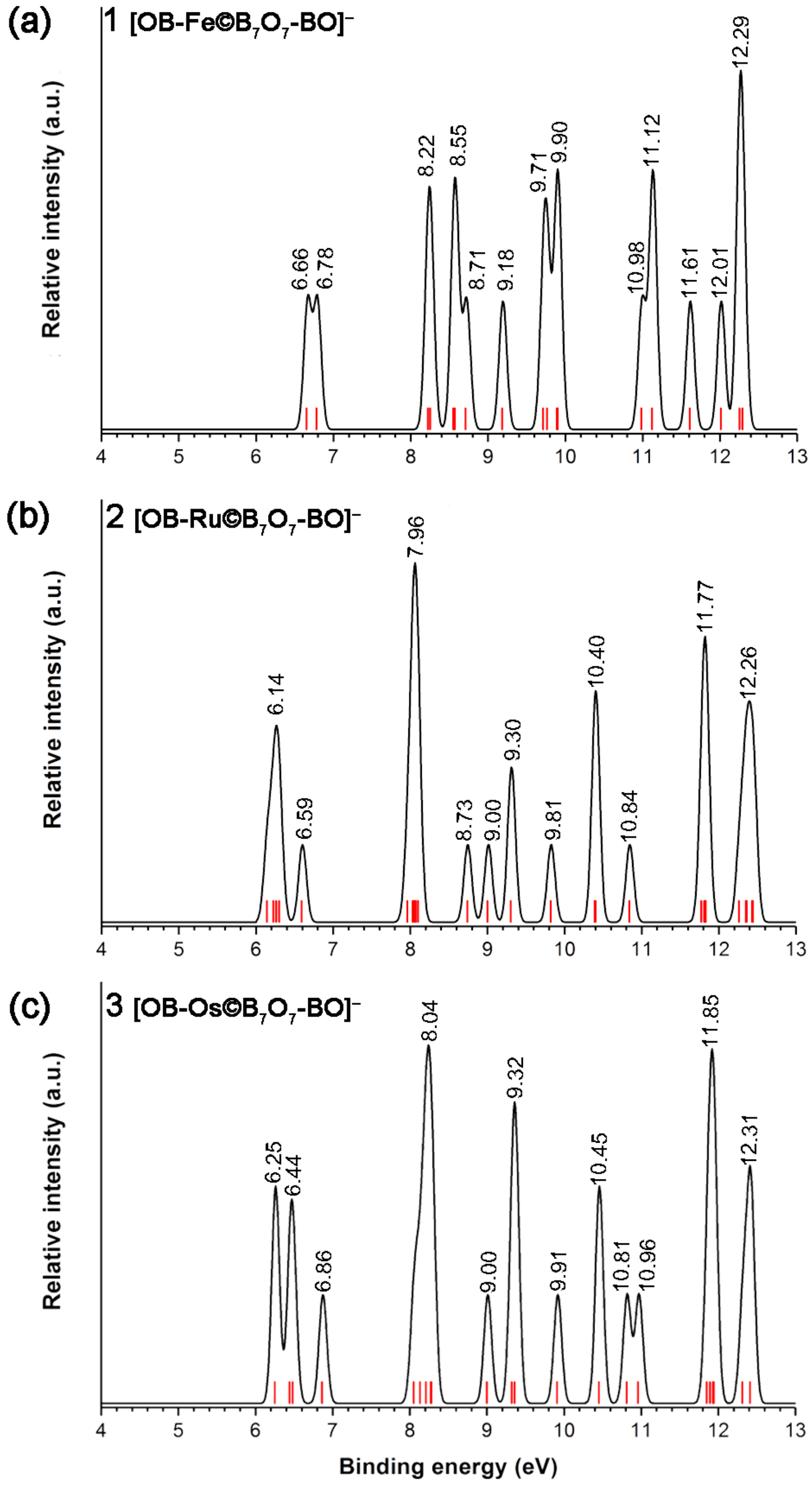

Supplementary Figure S5. The simulated PES spectra of 1–3 at the TD-PBE0/BS1 level. The first VDEs of **1–3** were corrected using CCSD(T)/BS1 calculations. The adsorption curves were simulated by fitting the calculated VDEs (vertical bars) using unit-area Gaussian function with half width of 0.1 eV.

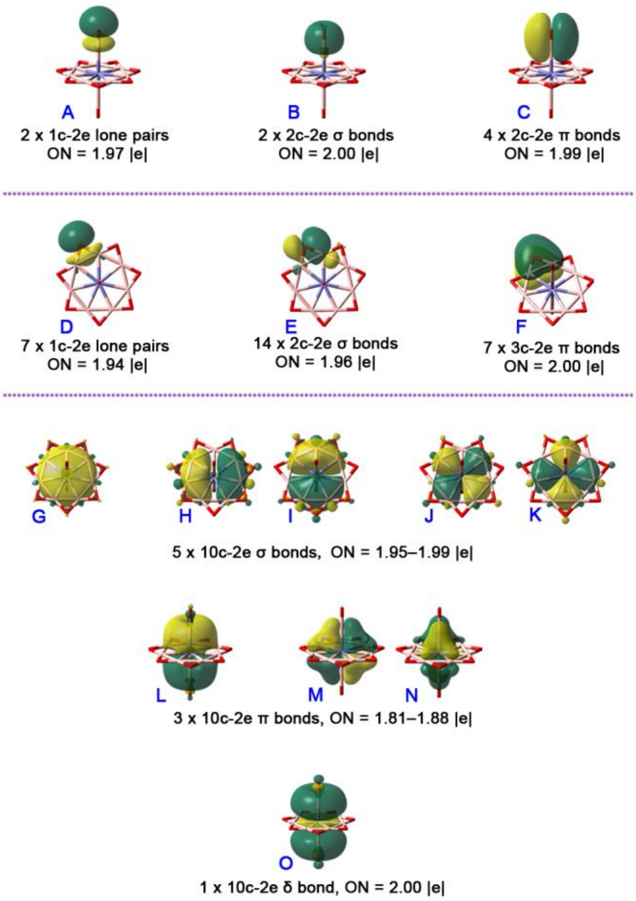

Supplementary Figure S6. AdNDP bonding patterns of [OB-Fe©B_7O_7-BO]$^-$ (1). Only one orbital is showed when multiple orbitals in a pattern are identical due to molecular symmetry.

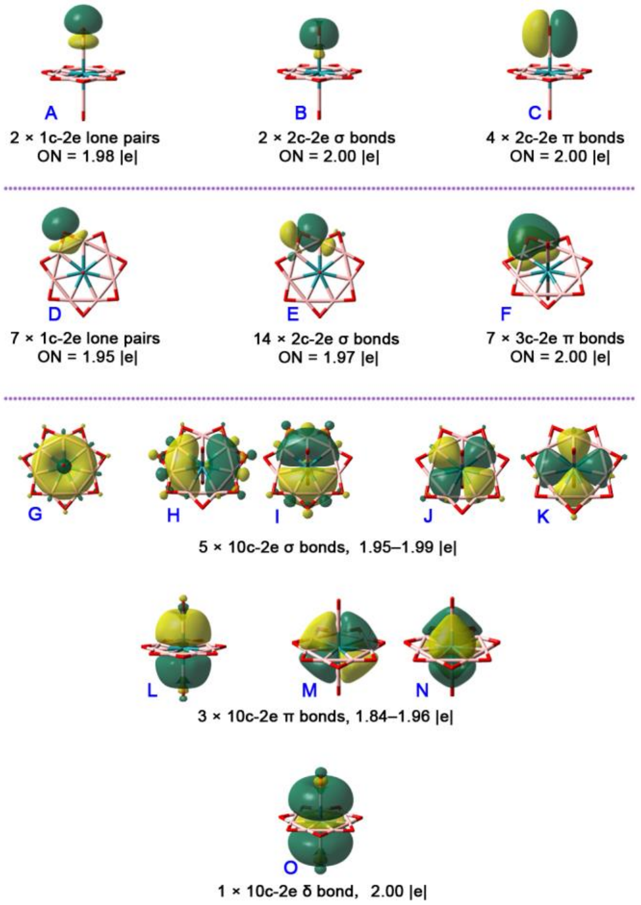

Supplementary Figure S7. AdNDP bonding patterns of [OB-Ru©B_7O_7-BO]$^-$ (2). Only one orbital is showed when multiple orbitals in a pattern are identical due to molecular symmetry.

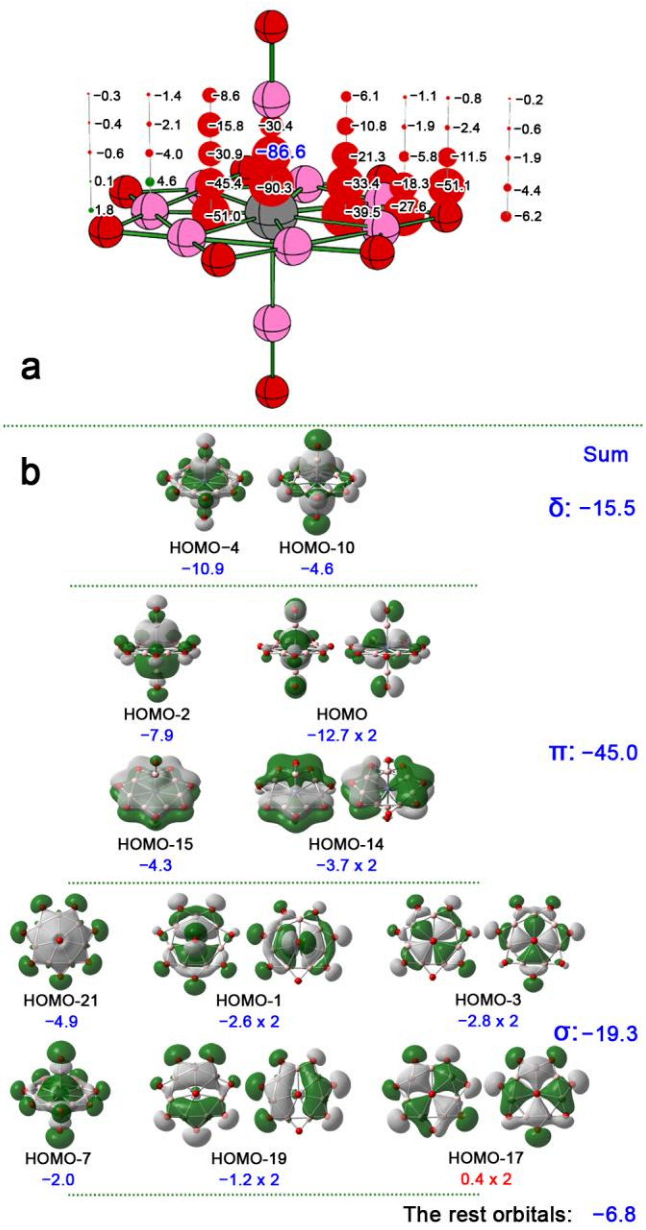

Supplementary Figure S8. NICS results of 1. **a** The calculated NICS values for the ghost atoms located in a symmetry plane (the positive and negative NICS values are shown in green and red bare balls, respectively). **b** The dissected contributions from individual canonical molecular orbitals to the total NICS value for the point located 1 Å above the center Fe atom.

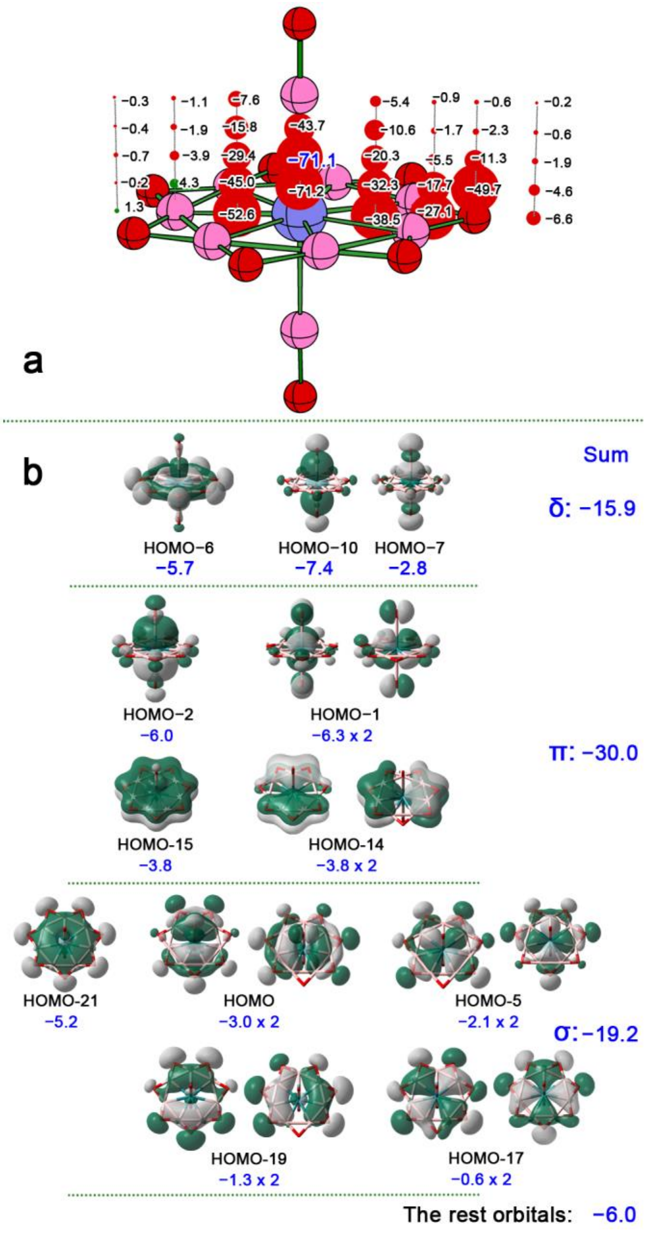

Supplementary Figure S9. NICS results of 2. **a** The calculated NICS values for the ghost atoms located in a symmetry plane (the positive and negative NICS values are shown in green and red bare balls, respectively). **b** The dissected contributions from individual canonical molecular orbitals to the total NICS value for the point located 1 Å above the center Ru atom.

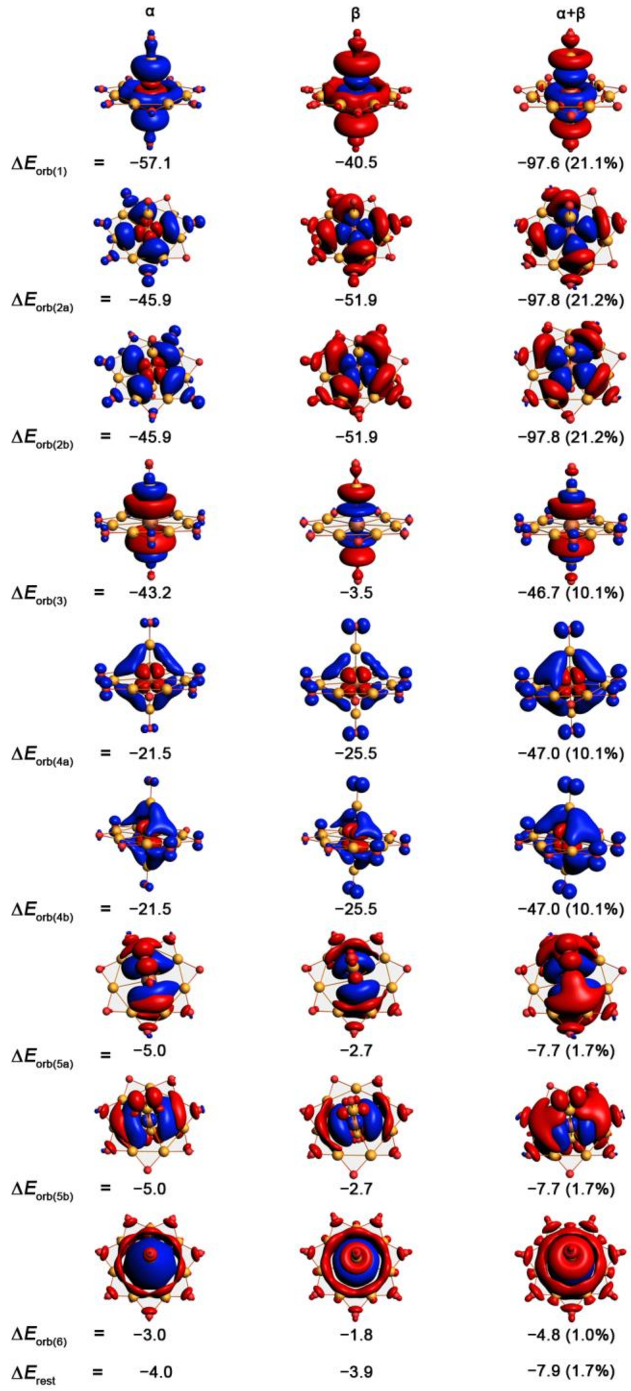

Supplementary Figure S10. The shapes of deformation densities ($\Delta\rho$) for EDA-NOCV analysis of 1. Both the related molecular orbitals and corresponding α and β spin orbitals are shown. The isovalues of the surfaces are 0.001 for $\Delta\rho_{\mathrm{orb}(1)\text{–}(4)}$, 0.0003 for $\Delta\rho_{\mathrm{orb}(5)}$ and 0.0002 for $\Delta\rho_{\mathrm{orb}(6)}$. The direction of charge flow is from red to blue.

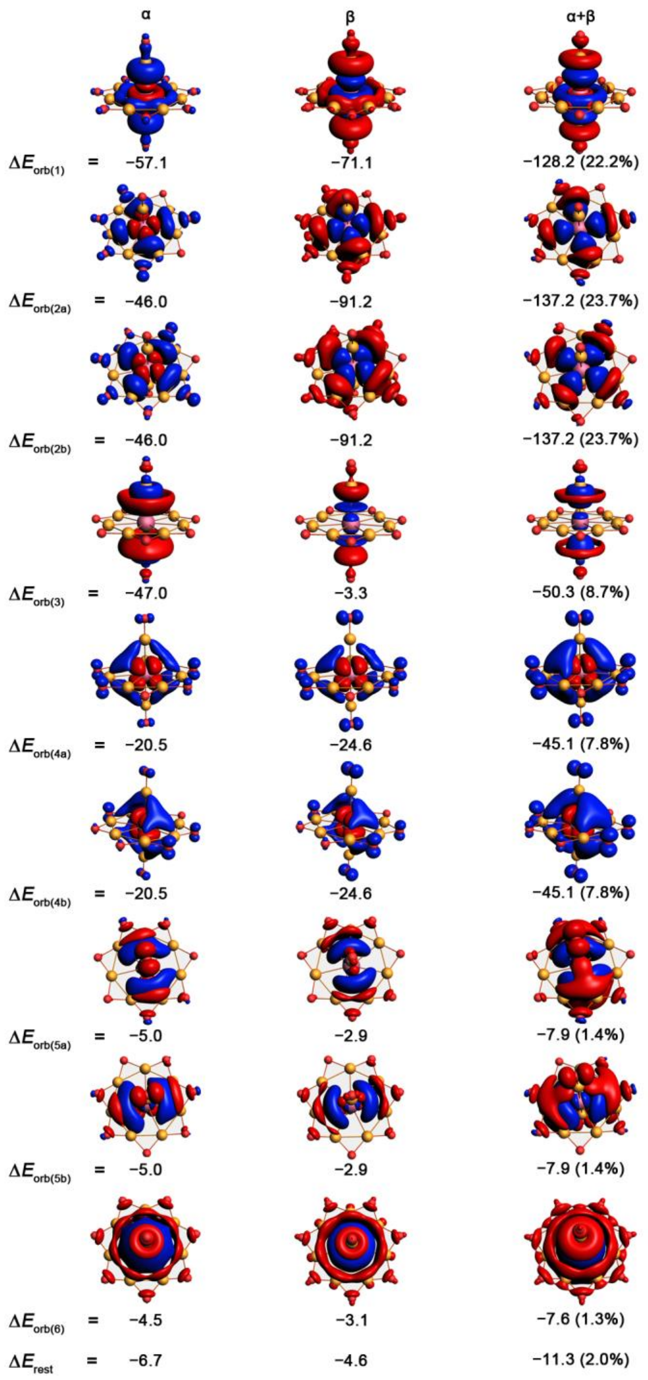

Supplementary Figure S11. The shapes of deformation densities ($\Delta\rho$) for EDA-NOCV analysis of 2. Both the related molecular orbitals and corresponding α and β spin orbitals are shown. The isovalues of the surfaces are 0.001 for $\Delta\rho_{\mathrm{orb(1)–(4)}}$, 0.0003 for $\Delta\rho_{\mathrm{orb(5)}}$ and 0.0002 for $\Delta\rho_{\mathrm{orb(6)}}$. The direction of charge flow is from red to blue.

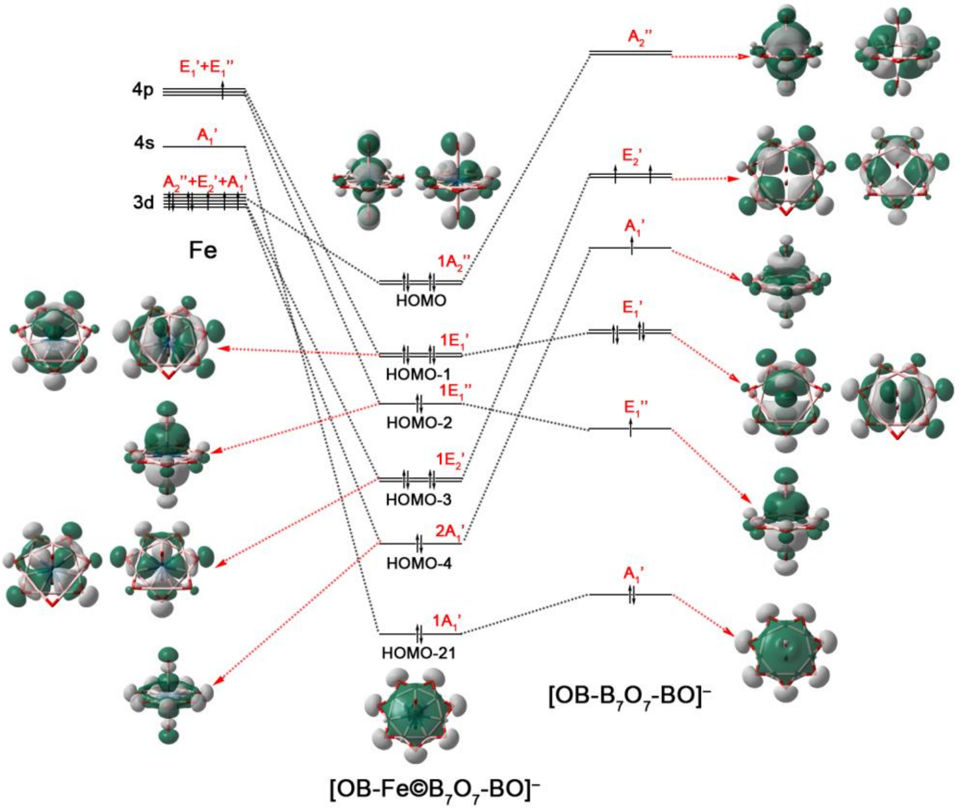

Supplementary Figure S12. MO correlation diagram of Fe with [OB-B$_7$O$_7$-BO]$^-$ ligand.

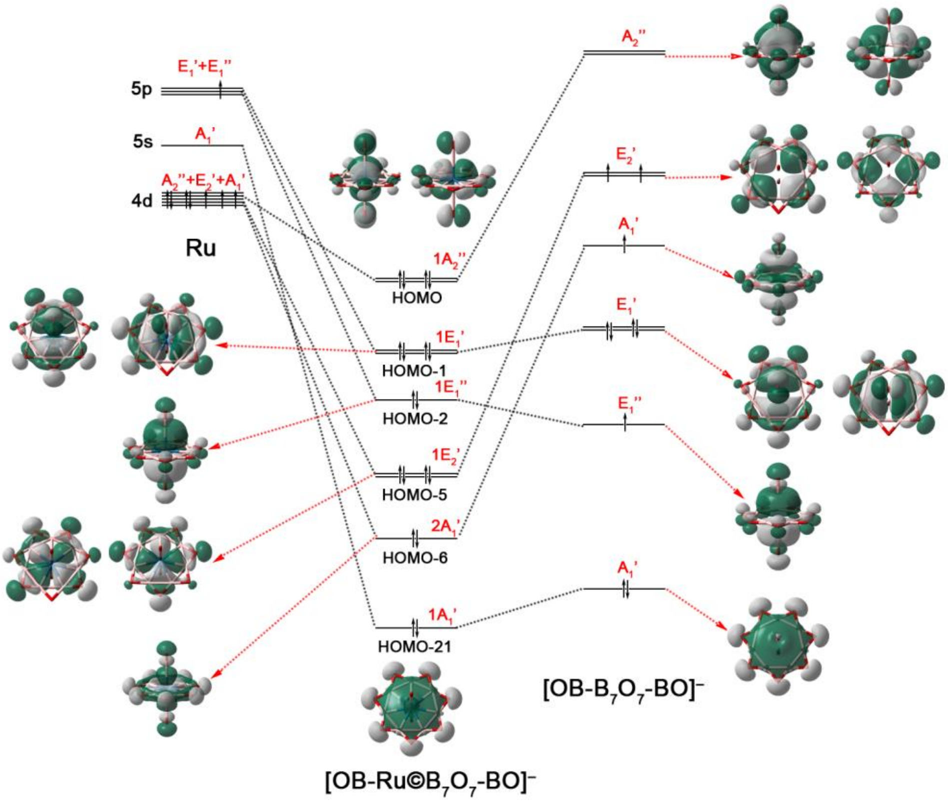

Supplementary Figure S13. MO correlation diagram of Ru with [OB-B$_7$O$_7$-BO]$^-$ ligand.

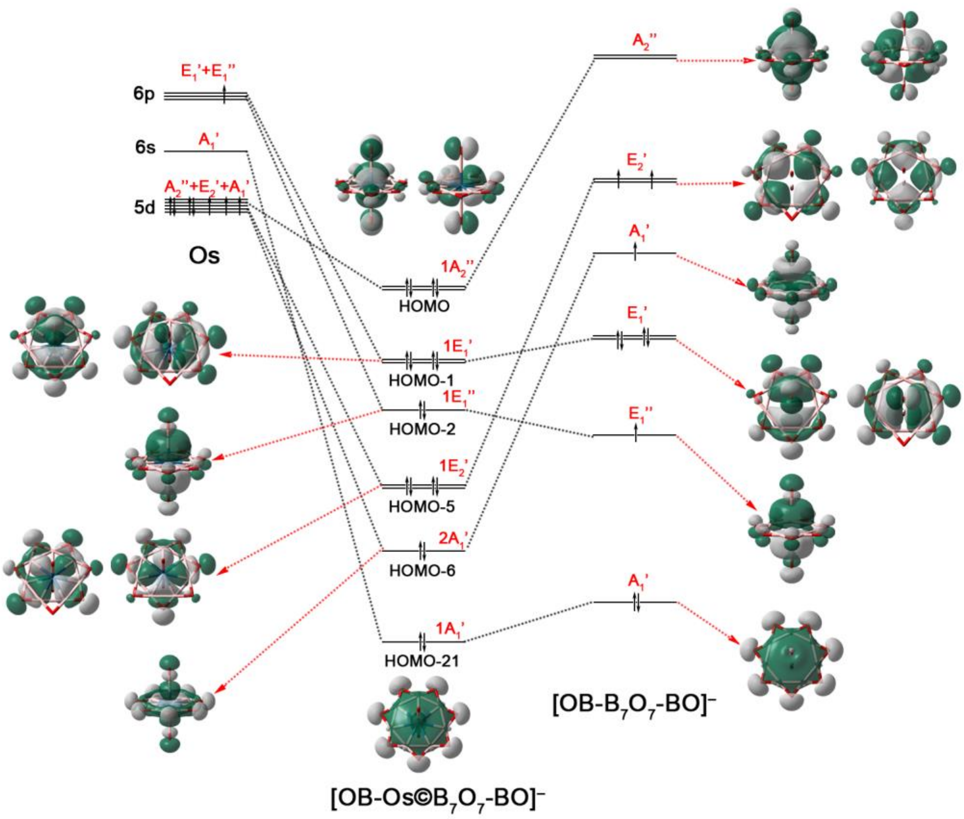

Supplementary Figure S14. MO correlation diagram of Os with $[OB\text{-}B_7O_7\text{-}BO]^-$ ligand.
