# Supplementary material for: Prediction of heptagonal bipyramidal nonacoordination in highly viable [OB-M©B7O7-BO]− (M = Fe, Ru, Os) complexes
Source: Commun Chem. 2022 Jan 10;5:1. doi: 10.1038/s42004-021-00620-0 (PMC9814638; doi:10.1038/s42004-021-00620-0)
Supplement: Supplementary file 3 — Description of Additional Supplementary Files [file 42004_2021_620_MOESM3_ESM.pdf]

Description of Additional Supplementary Files

File name: Supplementary Data 1
Description: Cartesian coordinates of concerned structures.
